# Supplementary material for: Progressive Evolutionary Dynamics of Gene-Specific ω Led to the Emergence of Novel SARS-CoV-2 Strains Having Super-Infectivity and Virulence with Vaccine Neutralization
Source: Int J Mol Sci. 2024 Jun 7;25(12):6306. doi: 10.3390/ijms25126306 (PMC11204377; doi:10.3390/ijms25126306)
Supplement: Supplementary file 1 [file ijms-25-06306-s001.zip › ijms-3022713-supplementary.pdf]

Supplementary data

Supplementary Table S1: Number of average mutations in alpha, delta and omicron strain in compared to original strain and number of mutations in reconstructed alpha, delta and omicron strain from 100 genomes of various part of the world that signifies the variations/divergence in the population.

**Strain**	**Average no of mutation**	**Mutations in 100 genomes of SARS-CoV-2**
Alpha	22.7	131 (Sept'2020)
delta	48.5	= (484+467)/2=475.5 (June' 2021, July'2021)
omicron	74.2	= (365+326+267+ 247)/4=301.25 (Jan, March, April, May 2022

Supplementary Table S2. Rate of change of over time and in major strain whole genome (WG) and spike protein

**Whole enome**									
months	Jan, 2020-0-months	ω-alpha-sept20-9 months	ω-delta-june21-18 months	ω-delta-july21-19 months	ω-omi-Jan22-25 months	ω-omi-March22-27 months	ω-omi-April22-28 months	ω-omi-May22-29 months	Avg of all months
ω	0.04	0.35	0.68	0.56	0.79	0.64	0.68	0.67	
avg rate (ω_{avg})	0	0.034	0.023	-0.120	0.71	-0.075	0.04	-0.01	0.025
avg rate (ω_{avg})/site/yr	-	1.382E-05	9.364E-06	-4.82E-05	0.00028	-3.01E-05	1.605E-05	-4.013E-06	1E-05
instant rate (dω/dt)	0	0.04	0.038	0.029	0.0316	0.024	0.024	0.023	0.03
instant rate (dω/dt)/site/yr		1.561E-05	1.516E-05	1.183E-05	1.3E-05	9.512E-06	9.746E-06	9.2714E-06	1.2E-05
**Spike**									
months	Jan, 2020-0-months	ω-alpha-sept20-9 months	ω-delta-june21-18 months	ω-delta-july21-19 months	ω-omi-Jan22-25 months	ω-omi-March22-27 months	ω-omi-April22-28 months	ω-omi-May22-29 months	Avg of all months
ω	0.02	0.15	1.24	0.64	1.97	1.97	1.07	1.43	
avg rate (ω_{avg})	0	0.01	0.12	-0.03	1.79	0	-0.9	0.36	0.15
ω_{avg}/site/yr		5.797E-06	4.86E-05	-1.27E-05	0.00072	0	-0.000361	0.00014447	6.191E-05
instant rate (dω/dt)	0	0.02	0.07	0.03	0.08	0.07	0.04	0.05	0.05

instant rate (dω/dt)/site/yr		6.69E-06	2.764E-05	1.352E-05	3.2E-05	2.928E-05	1.534E-05	1.9788E-05	2.233E-05

Supplementary Table S3: List of SARS-CoV-2 genes associated for various functions.

	**SARS-CoV-2 replication and multiplication genes**	**Packaging and virus assembly genes**	**Host immunity Evading/escaping genes**
**Genes/protein**	NSP12/RDRP, NSP3, NSP4, NSP7, NSP9 NSP13, NSP8, Orf9	Orf4, NSP14, Orf3a, Orf7b	NSP1, Spike, NSP10, NSP16, Orf3a, Orf8, Orf10, NSP5, Orf6, Orf7a

Supplementary Table S4. List of genes in increased, decreased or unchanged in various isolates.

ω	Alpha	Delta	Omicron
**Highest/increased**	**NSP2**	**RDRP/NSP12, Orf5, NSP16, NSP10, Orf8, NSP8, Orf6, Orf6, Orf7a**	**Spike, NSP4, Orf9, NSP1, NSP3, NSP7, NSP9, NSP14, NSP15, Orf3a, NSP5, NSP13 Orf7a, Orf7b**
**Decreased**		**NSP2**	**NSP2**
**Unchanged**	**NSP6, Orf10**	**NSP6, Orf10**	**NSP6, Orf10**

Supplementary Table S5: Important nonsynonymous mutations of SARs-CoV2 genes affecting functions and phenotypes

Gene Name	Mutations	Alpha	Delta	omicron	phenotypes
Spike	D614G	+	+	+	Super infectivity[1]
	N501Y	+	+	+	Increased infectivity
	L452R		+		Deficiency in antibody neutralization and transmission
	E484Q		+		Deficiency in antibody neutralization and transmission [2]
	P681R				Increased transmission
NSP1	R24C, D75E	+	?	?	Increased flexibility[3]
NSP2	G262V/G265V, E63K/E66K, K330D/K337D	+			Reduced interaction p450 reductase leads to increased 40s-ribosome interaction[4]
NSP3	P822L, V765F, G255V, A488S		+		Increased replication, virulency and drug resistance[5]
NSP4	T492I		+		Increased replication, virulency and drug resistance[5]
	M324				[6]
NSP5	N65S		+		Increased replication, virulency and drug resistance [5]
NSP6	ΔSGF (3 aa deletion)	+			Increased replication assembly by inducing ER zippering (gain of function)[7]
NSP7	S26F/A, I68T/V, R79S/G	+			Increase of RDRP-NSP7-NSP8 complex replication efficiency[8]
NSP8	M129I, I156V	+			Increase of RDRP-NSP7-NSP8 complex

					replication efficiency[8]
NSP9					No significant mutations are characterized yet.
NSP10	A20V,		+		Alterations of NSP10-NSP16 intramolecular complex formation[9]
NSP12/ RDRP	P323L	+	+	+	Increased replication[10]
	A97V	+	+	+	Decreased replication fidelity[11]
NSP13	P504L, Y541C	+	+		Increased fatality rates[12]
NSP14	F233L				Increased proof reading activities during replication [13]
NSP15	H234Y S261L		+		viral oligomerization and increased viral replication[5]
NSP16	P236L		+		Alterations of NSP10-NSP16 intramolecular complex formation[9]
Orf3a	G251V, Q57H G172V				Destabilizing its own structure[14, 15]
Orf4	P71L	+	+		Increased stability leading to transport from Golgi to ER[16]
Orf5	L54F, Q91E				Highly destabilizing its own structure[14], increases fitness[17]
Orf6					
Orf7a	X122L				Deleterious or neutral (X due to extension of ORF at stop codon)[14]
Orf7b					
Orf8	382nt deletion	+			Reduced cytokine storm, inflammation and milder symptoms
	S24L		+		Increases folding stability (highly female dominated mutation, gender bias)[18]

Orf9	R203K/G204 R	+			Increased replication efficiency
Orf10	P10S, I4V	+	+		Increased binding activities but no change in immunogenicity[19]

Reference

1. Long, S.W., et al., *Molecular Architecture of Early Dissemination and Massive Second Wave of the SARS-CoV-2 Virus in a Major Metropolitan Area.* mBio, 2020. **11**(6).
2. Mohammadi, M., M. Shayestehpour, and H. Mirzaei, *The impact of spike mutated variants of SARS-CoV2 [Alpha, Beta, Gamma, Delta, and Lambda] on the efficacy of subunit recombinant vaccines.* Braz J Infect Dis, 2021. **25**(4): p. 101606.
3. Mou, K., et al., *Emerging Mutations in Nsp1 of SARS-CoV-2 and Their Effect on the Structural Stability.* Pathogens, 2021. **10**(10).
4. Gupta, M., et al., *CryoEM and AI reveal a structure of SARS-CoV-2 Nsp2, a multifunctional protein involved in key host processes.* bioRxiv, 2021.
5. Sahni , C., et al., *SARS-CoV-2 Mutations Responsible for Immune Evasion Leading to Breakthrough Infection <p class="MsoNormal" style="margin-top:2.4pt;margin-right:12.0pt;margin-bottom:*

6.0pt;margin-left:0in;line-height:15.6pt;mso-outline-level:1;background:white">. 2022: Cureus. p. e29544.

6. Vilar, S. and D.G. Isom, *One Year of SARS-CoV-2: How Much Has the Virus Changed?* Biology (Basel), 2021. **10**(2).
7. Ricciardi, S., et al., *The role of NSP6 in the biogenesis of the SARS-CoV-2 replication organelle.* Nature, 2022. **606**(7915): p. 761-768.
8. Reshamwala, S.M.S., et al., *Mutations in SARS-CoV-2 nsp7 and nsp8 proteins and their predicted impact on replication/transcription complex structure.* J Med Virol, 2021. **93**(7): p. 4616-4619.
9. Azad, G.K., *Identification of novel mutations in the methyltransferase complex (Nsp10-Nsp16) of SARS-CoV-2.* Biochem Biophys Rep, 2020. **24**: p. 100833.
10. Ilmjärv, S., et al., *Concurrent mutations in RNA-dependent RNA polymerase and spike protein emerged as the epidemiologically most successful SARS-CoV-2 variant.* Sci Rep, 2021. **11**(1): p. 13705.
11. Eskier, D., et al., *RdRp mutations are associated with SARS-CoV-2 genome evolution.* PeerJ, 2020. **8**: p. e9587.
12. Cao, C., et al., *Molecular epidemiology analysis of early variants of SARS-CoV-2 reveals the potential impact of mutations P504L and Y541C (NSP13) in the clinical COVID-19 outcomes.* Infect Genet Evol, 2021. **92**: p. 104831.
13. Eskier, D., et al., *Mutations of SARS-CoV-2 nsp14 exhibit strong association with increased genome-wide mutation load.* PeerJ, 2020. **8**: p. e10181.
14. Vassilaki, N., et al., *SARS-CoV-2 Amino Acid Mutations Detection in Greek Patients Infected in the First Wave of the Pandemic.* Microorganisms, 2022. **10**(7).

15. Tasakis, R.N., et al., *SARS-CoV-2 variant evolution in the United States: High accumulation of viral mutations over time likely through serial Founder Events and mutational bursts.* PLoS One, 2021. **16**(7): p. e0255169.
16. Singh, J., et al., *Structure-Function Analyses of New SARS-CoV-2 Variants B.1.1.7, B.1.351 and B.1.1.28.1: Clinical, Diagnostic, Therapeutic and Public Health Implications.* Viruses, 2021. **13**(3).
17. Obermeyer, F., et al., *Analysis of 6.4 million SARS-CoV-2 genomes identifies mutations associated with fitness.* Science, 2022. **376**(6599): p. 1327-1332.
18. Wang, R., et al., *Analysis of SARS-CoV-2 mutations in the United States suggests presence of four substrains and novel variants.* Commun Biol, 2021. **4**(1): p. 228.
19. Mishra, S., *Computational Structural and Functional Analyses of ORF10 in Novel Coronavirus SARS-CoV-2 Variants to Understand Evolutionary Dynamics.* Evol Bioinform Online, 2022. **18**: p. 11769343221108218.

Supplementary Table S6: R_0 estimation in original, alpha and delta strain in Iranian population

Methods	Original strain	alpha	delta	
Exponential Growth Rate (EGR)	0.99	2.26	3.0,	[1]
Maximum Likelihood (ML)	1.04	2.64	3.1	
Sequential Bayesian (SB)	1.06	11.38	12	
time-dependent SIR	2.79	12.13	23.13	
Computer modelling	2.79	--	5.08	[2]

References

1. Sheikhi, F., et al., *Estimation of the basic reproduction number of Alpha and Delta variants of COVID-19 pandemic in Iran.* PLoS One, 2022. **17**(5): p. e0265489.
2. Liu, Y. and J. Rocklöv, *The reproductive number of the Delta variant of SARS-CoV-2 is far higher compared to the ancestral SARS-CoV-2 virus.* J Travel Med, 2021. **28**(7).

Supplementary Table S7: The SARS-CoV-2 genome accession no that are used for mutation identification

January 2020

MN908947

September 2020

MW035476, MW040645, MW035558, MW035556, MW035570.1, MW077468, MW077477, MW070096.1,
MW035542, MW035466.1, MW070107.1, MW070096, MW075732, MW031044, MW155352, MW155192,
MW030990, MW031006, MW035532, MW157210, MW185627, MW155442, MW155194, MW030992,
MW075751, MW155198, MW155185, MW157210, MW157208, MW157205, MW075768, MW075757.1
MW075760, MW075763, MW031002, MW155334, MW155301 MW990314 MW990318 MW990330
OD928954, OD916524, OD928954, OD938526, OB997770, OB997810, OB998345, MW972889 MW972902, MW972904,
MW972909, MW973393, OA982885, OA982892, OA982904, OA982913,
OA982924, OA982930, OA982947, OA982956, OA982975, OA983055, OA983060, OA983062,
OA983068, OA983089, OA983100, MW309426, MW309429, MW306374, MW306379, MW306403,
MW306439, MW306442, MW306446, MW276241, MW276313, MW276578, LR898893, MW280547,
LR898893, MW280548, MW280534, MW280528, MW280523, MW280494, MW280525, MW280486,
LR898896, LR898900, LR898906, LR898911, LR898915, LR898925, LR898936, LR898962,

MW269882, MW269890, MW269893, MW269903, MW268711, MW268717, MW268726, MW269898

June 2021

ON703255.1, ON647472.1, ON618530.1, ON601203.1, ON601205.1, ON601208.1, ON486697.1, ON486703.1, ON486711.1, ON486725.1, ON486727.1, ON486837.1, ON430744.1, ON440192.1, ON440224.1, ON430744.1, ON440192.1, ON424662.1, ON415787.1, OW667328.1, OW674895.1 OW676658.1, ON338104.1, ON338105.1, ON338112.1, ON322592.1, ON322618.1, ON322622.1, ON322683.1, ON322684.1, ON322685.1, ON322690.1, ON298112.1, ON287354.1, ON304577.1, ON304578.1, ON304594.1, ON282189.1, ON282192.1, ON282197.1, ON286651.1, OW470290.1, OW471457.1, OW469301.1, OW471228.1, OW495332.1, OW496392.1, OW501739.1, OW502870.1 OW503386.1, OW503946.1, OW512632.1, LC654313.1, OW443685.1, OW443723.1, OW443729.1, OW455053.1, OW456758.1, OW457077.1, ON213099.1, ON213129.1, ON213149.1, ON213161.1, ON213168.1, ON213172.1, ON213172.1, ON213173.1, ON213185.1, ON215686.1, ON184758.1, ON184759.1, ON184767.1, ON184808.1, ON184816.1, ON184859.1, ON184954.1, ON184979.1, OW387566.1, OW388391.1, OW412478.1, OW416467.1, ON168577.1, ON168668.1, ON150683.1 ON078406.1, ON080874.1, ON074378.1, OW094160.1, OW094296.1, OW095167.1, OW094827.1, OW098566.1

July 2021

ON485295.1, ON485437.1, ON485439.1, ON486744.1, ON439127.1, ON439134.1, ON439151.1, ON439159.1 ON439169.1, ON440090.1, ON440099.1, ON454049.1, ON454051.1, ON454065.1, ON454356.1, ON454379.1 ON411389.1, ON412861.1, ON412878.1, OW677549.1, OW670514.1, OW668674.1, OW667600.1, OW611228.1 ON364571.1, OW520733.1, OW522557.1, OW522883.1, OW523101.1, OW523117.1, ON292113.1, ON280180.1 ON280215.1, ON280382.1, ON280399.1, ON280416.1, ON280421.1, ON280442.1, ON280474.1, ON280588.1 ON284344.1, OW443723.1, OW443729.1, OW456193.1, ON213081.1, ON215616.1, ON199092.1, ON199102.1 ON199107.1, ON199110.1, ON199124.1, ON186017.1, ON186056.1, ON186058.1, ON186062.1, OW404193.1 ON131019.1, ON131025.1, ON131162.1, OW319147.1, OW332191.1, OW332263.1, OW333584.1, ON099808.1 ON099811.1, ON099817.1, ON099820.1, ON099822.1, ON099824.1, ON099826.1, ON099829.1, ON099832.1 ON099914.1, ON099925.1, ON099970.1, ON099974.1, OW312745.1, OW312799.1, OW314361.1, ON030921.1 ON030922.1, ON030923.1, ON017304.1, ON017626.1, ON017629.1, ON017640.1, ON017661.1, ON008257.1 ON008250.1, ON008286.1, ON008288.1, OM961186.1, OM922485.1, OM922532.1, OM922777.1, OM922779.1, OM922788.1, OM899726.1, OM913722.1 OM913728.1, OM899727.1

January 2022

ON382452.1, ON382461.1, ON382464.1, ON369498.1, ON369503.1, ON369507.1, ON356307.1
ON356307.1, ON332944.1, ON338419.1, ON326638.1, ON327305.1, ON327305.1, ON328162.1
ON328162.1, ON323327.1, ON323335.1, ON316381.1, ON262464.1, ON262464.1 , ON262480.1
ON262537.1, ON263622.1, ON263852.1, ON264247.1, ON264316.1, ON276619.1, ON238389.1
ON222989.1, ON222966.1, ON222971.1, ON222989.1, ON222993.1, ON222995.1, ON214967.1
ON214968.1, ON215014.1, ON215020.1, ON215044.1, ON215063.1, ON215075.1, ON215207.1
ON215244.1, ON215260.1, ON215267.1, ON220952.1, ON382451.1, ON215011.1, ON215307.1
ON215307.1, ON304735.1, ON356281.1, ON359522.1, ON359522.1, ON265942.1, ON266015.1
ON265997.1, ON266052.1, ON259981.1, ON316396.1, ON265952.1, ON265981.1, ON316425.1
ON220939.1, ON271492.1, ON269568.1, ON558126.1, ON558120.1 ON558153.1, ON558188.1
ON558198.1, ON558202.1, ON558227.1, ON558241.1, ON558275.1, ON558322.1, ON558330.1
ON558472.1, ON558521.1, ON558525.1, ON558531.1, ON558582.1, ON558611.1, ON558613.1
ON558629.1, ON558637.1, ON558677.1, ON558692.1, ON559088.1, ON559164.1, ON559291.1
ON559708.1, ON560532.1, ON550079.1, ON550087.1, ON550090.1, ON550253.1, ON554547.1
ON554614.1, ON554611.1, ON554620.1, ON554687.1, ON554689.1, ON554690.1, ON554705.1
ON554727.1, ON554736.1, ON554742.1, ON554742.1, ON554747.1, ON554938.1, ON555015.1
ON555018.1, ON555974.1, ON555462.1, ON555986.1, ON555997.1, ON555997.1, ON556004.1
ON556020.1

March 2022

ON384055.1, BS004211.1, BS004206.1, BS004207.1, ON333064.1, ON333065.1, ON384051.1
ON384088.1, ON384080.1, ON384286.1, ON384307.1, OM988444.1, OM988448.1, OM988452.1
OM988458.1, OM988459.1, OM988469.1, OM988471.1, OM988474.1, OM988482.1, OM988486.1
OM988494.1, OM988497.1, OM988502.1, OM988504.1, OM988520.1, OM988528.1, OM988564.1,
OM988575.1, OM988585.1, OM988596.1, OM988598.1, OM988599.1, OM988614.1, OM988623.1
OM988633.1, OM988634.1, OM988639.1, OM988644.1, OM988654.1, OM988661.1, OM988662.1,
OM988664.1, OM988871.1, OM988872.1, OM988881.1, OM988888.1, OM988898.1, OM988910.1
OM988915.1, OM988959.1, OM988962.1, OM988991.1, OM989002.1, OM989009.1, OM989012.1
OM989015.1, OM989024.1, OM989025.1, OM989038.1, OM989040.1, OM989047.1, OM989049.1,

OM989055.1, OM989058.1, ON558486.1, ON558575.1, ON559386.1, ON560473.1, ON560672.1,
ON547699.1, ON528758.1, ON528761.1, ON528779.1, ON528822.1, ON528841.1, ON532424.1
ON533354.1, ON518911.1, ON518916.1, ON518951.1, ON518962.1, ON518978.1, ON518980.1
ON519004.1, ON519015.1, ON519015.1, ON519015.1, ON519020.1 ON519036, ON519048.1
ON519049.1, ON521655.1, ON521658.1, ON521664.1, ON521678.1, ON521679.1, ON521679.1,
ON521690.1, ON521701.1, ON524952.1, ON525866.1, ON525873.1, ON525982.1, ON525997.1
ON526021.1, ON526029.1, ON526059.1, ON526071.1, ON526086.1, ON526077.1, ON526106.1
ON526117.1, ON526118.1, ON512088.1, ON496360.1, ON496217.1, ON502812.1, ON505456.1

April 2022

ON389299.1, ON389713.1, ON389744.1, ON389809.1, ON389920.1, ON389923.1, ON390000.1
ON390020.1, ON390049.1, ON390055.1, ON390103.1, ON390128.1, ON390131.1, ON390138.1
ON390158.1, ON390174.1, ON390177.1, ON390182.1, ON390186.1, ON390189.1, ON390190.1
ON390192.1, ON390395.1, ON390428.1, ON390428.1, ON390433.1, ON390501.1, ON390574.1
ON390588.1, ON390653.1, ON390710.1, ON390716.1, ON390744.1, ON390748.1, ON390757.1
ON390758.1, ON390770.1, ON390772.1, ON390781.1, ON391485.1, ON391486.1, ON391489.1,
ON391489.1, ON392553.1, ON392561.1, ON392565.1, ON392573.1, ON392574.1, ON392578.1
ON393193.1, ON393195.1, ON393199.1, ON393204.1, ON393221.1, ON393222.1, ON393228.1,
ON393231.1, ON393232.1, ON393232.1, ON393237.1, ON393241.1, ON393244.1, ON393243.1
ON521735.1, ON521744.1, ON526120.1, ON526129.1, ON528912.1, ON528916.1, ON528917.1,
ON528918.1, ON528920.1, ON531819.1, ON531822.1, ON531824.1, ON531836.1, ON531884.1
ON531909.1, ON541044.1, ON541048.1, ON541057.1, ON541057.1, ON548740.1, ON548743.1
ON548763.1, ON549929.1, ON549932.1, ON549938.1, ON549943.1, ON549944.1, ON549948.1
ON549951.1, ON549954.1, ON549958.1, ON549965.1, ON549965.1, ON562700.1, ON562703.1
ON562740.1, ON562749.1, ON562763.1, ON562770.1, ON562800.1, ON562827.1, ON562842.1,
ON562848.1, ON562870.1, ON562871.1, ON562886.1, ON562888.1, ON562908.1, ON562912.1,
ON562916.1

May 2022

ON505844.1, ON505845.1, ON505852.1, ON505855.1, ON509609.1, ON509607.1, ON509607.1, ON509607.1
ON509615.1, ON509618.1, ON510068.1, ON510058.1, ON510055.1, ON510062.1, ON510067.1, ON510194.1,

ON510195.1, ON510198.1, ON510200.1, ON510203.1, ON510208.1, ON510210.1, ON510221.1, ON510225.1
ON510227.1, ON510230.1, ON510232.1, ON510252.1, ON510255.1, ON510260.1, ON510263.1, ON510266.1
ON510267.1, ON496025.1, ON496025.1, ON496032.1, ON496085.1, ON496147.1, ON499779.1, ON499780.1
ON499784.1, ON499785.1, ON499787.1, ON499790.1, ON499792.1, ON499798.1, ON499802.1, ON499804.1
ON499808.1, ON499811.1, ON499813.1, ON499814.1, ON499815.1, ON499816.1, ON499818.1, ON499820.1
ON499822.1, ON499822.1, ON499826.1, ON499830.1, ON499831.1, ON499833.1, ON499835.1, ON499840.1
ON499842.1, ON499843.1, ON499844.1, ON499847.1, ON499849.1, ON499852.1, ON499853.1, ON499857.1
ON499858.1, ON499862.1, ON499870.1, ON499873.1, ON499875.1, ON499876.1, ON499883.1, ON499885.1
ON499888.1, ON499892.1, ON499893.1, ON499894.1, ON499895.1, ON499898.1, ON499900.1, ON499907.1
ON499908.1, ON499912.1, ON499919.1, ON499921.1, ON499923.1, ON499927.1, ON499931.1, ON499942.1
ON499956.1, ON499965.1, ON499973.1, ON499982.1, ON499983.1, ON500001.1, ON500006.1, ON500012.1
ON500048.1, ON500049.1
